# Retraction: Colonic Flora, Probiotics, Obesity and Diabetes

**DOI:** 10.3389/fendo.2013.00110

**Published:** 2013-08-20

**Authors:** Frontiers in Endocrinology Editorial Office

The corresponding author (Paul E. Marik) and the journal wish to retract the 11 July 2012 article cited above.

While all parties are convinced of the value of this work, this Mini Review was found to contain text that was improperly cited.

We regret this inconvenience and apologize to the readers of Frontiers in Endocrinology.

